# On the Hydrostatic Test

**Published:** 1850-08

**Authors:** 


*On the Hydrostatic Test.* By Dr. Casper.—Dr. Casper states, that the very numerous opportunities he has had of investigating suspicious deaths, enable him to protest against the doubts which have been thrown upon the validity of this test. Of what value is the objection derived from the possibility of *emphysema pulmonum* being present, for who has ever seen a pathological emphysema in a new-born child? So, too, in speaking of the possibility of *artificial inflation*, we forget the nature of the cases which practically come before us. They are examples of solitary and clandestine delivery; and who is to act the perfidious part of infiltration? Moreover, every one who has tried it for himself must know how difficult it is to fill the lungs of a new-born child with air, and only once in about ten minutes can it be accomplished to the extent sufficient to alter the indications of the test. The objection derived from the effects of *putrefaction* is more important, but the careful judicial physician will never be deceived even here. It results from the author's repeated investigations, that the lungs are among the organs which latest undergo the process of putrefaction; and we may with certainty declare, that if lungs taken from a fresh or only slightly putrified body swim, they do not do so in consequence of putrefaction. Even when the child and its lungs are in an advanced state of putrefaction, the test my be of value when it furnishes a *negative indication;* e. g. the lungs sinking, even when far advanced in putrefaction, as Dr. Casper has frequently observed them to do. A remarkable case occurred to him, in which the heart of a very putrid child swam, but in which the lungs sank. As to *atelectasis*, it is very rarely met with to a great extent in new-born children; and when it has been said to have been so, this has arisen from the products of inflammation, which, unaided by the microscope, may easily happen, being mistaken for it. The author exhibited to his class the lungs of a child who died when eight days old from pneumonia, and which, throughout, were of the brownish-red color and compact consistence of those of a dead-born child, and even the smallest portions of them sank in water. It is true that we very frequently meet with small isolated patches of atelectasis in the lungs of new-born children, but these should exert no influence in our appreciation of the test.—*Casper's Wochenscrift*, 1849, No. 47.

